# Editorial: Towards 2030: sustainable development goal 8: decent work and economic growth. A sociological perspective

**DOI:** 10.3389/fsoc.2024.1487233

**Published:** 2024-09-16

**Authors:** Andrzej Klimczuk, Delali A. Dovie, Minela Kerla, Magdalena Klimczuk-Kochańska, Piotr Toczyski

**Affiliations:** ^1^Department of Social Policy, Collegium of Socio-Economics, SGH Warsaw School of Economics, Warsaw, Poland; ^2^Centre for Ageing Studies, College of Humanities, University of Ghana, Accra, Ghana; ^3^The Association of Online Educators, Sarajevo, Bosnia and Herzegovina; ^4^Faculty of Management, University of Warsaw, Warsaw, Poland; ^5^Media and Communication Department, Institute of Philosophy and Sociology, The Maria Grzegorzewska University, Warsaw, Poland

**Keywords:** SDG8, unemployment, labor-market issues, social insurance, working poor, precarity, remote work

## Overview

This Research Topic explores Sustainable Development Goal (SDG) eight, which is to “promote sustained, inclusive, and sustainable economic growth, full and productive employment, and decent work for all.” It highlights the COVID-19 pandemic's severe impact and triggered global economic recession, worsened gender pay gaps, increased undeclared employment, and significantly raised unemployment (United Nations, 2024). From a sociology-specific perspective, this Research Topic examines the global and local implementation of SDG8, its adaptation to different geographical contexts, stakeholder involvement, and issues related to decent work conditions worldwide.

The Research Topic was edited in cooperation with two journals: “Frontiers in Sociology” and “Frontiers in Public Health.” The Research Topic contains ten articles by 38 authors in: Bangladesh, China, Colombia, Germany, India, Indonesia, Peru, the Republic of Korea, South Africa, Switzerland, and the United States. Four types of articles are included: seven original research articles (Cho et al.; Islam and Hoque; König and Seifert; Naveen et al.; Quispe Mamani et al.; Ran and Zhao; Vélez-Rolón et al.), one review article (Chigbu and Nekhwevha), one perspective article (Broecher and Painter), and one opinion article (Setiawan et al.). This Research Topic deals with, among others, telework, digital skills, education, entrepreneurship, gig economy, intellectual capital, and work-life balance. The Research Topic of articles is organized according to three themes.

## Theme I: transformations in the modern workforce

The papers start with Chigbu and Nekhwevha, who focus on promoting decent work and sustainable economic growth within SDG8. The authors argue that this goal requires addressing gender inequality, market economy consequences, informal sector roles, and environmental sustainability. The presented research critically reviews 108 papers, revealing persistent gender biases, income disparities, insecure working conditions, and insufficient support for informal workers, highlighting gaps in achieving inclusive and sustainable growth. The findings emphasize the importance of fair, safe, and secure employment opportunities to support economic growth and uphold workers' rights. In the following article, Cho et al., based on a South Korean case, introduce recent changes related to home-based telework that has changed rapidly due to the COVID-19 pandemic, necessitating adjustments in productivity, evaluations, and employment rules as per the Korean Labor Standards Act. This study underlines the need for societal and institutional shifts, including legal and organizational changes, to sustain post-pandemic remote work and optimize its dynamics for future advancements. König and Seifert continue discussing this subject by focusing on digital skills. Support for their development has become even more critical during the pandemic, especially for older employees working from home, although many still have lower digital proficiency. This research found that, among others, older workers expanded their computer skills during the pandemic. Ran and Zhao focus on another unexpected pandemic effect: the growing importance of occupational injury protection for gig workers in China. The authors show that the current measures are insufficient due to their exclusion from traditional employee benefits. This study contributes ideas for reforming work-related injury insurance to better protect gig workers, offering insights that may also benefit other countries.

## Theme II: challenges of bridging gaps between education and employment

Islam and Hoque's study on the trade-off between schooling and labor for children in rural Bangladesh opens the next section. This research finds that subsistence needs, labor demand, and parental occupation influence decisions to prioritize work over education. Additionally, factors such as sexual division of labor, credit constraints, and cultural beliefs negatively impact parents' decisions regarding child schooling, suggesting that interventions must consider these socio-economic and cultural factors. The team of Vélez-Rolón et al., in their research, focused on another example of the relationship between education and employment. The authors argue that rapid technological advances and global challenges, including the COVID-19 pandemic, underscore the need for educational models that enhance employability and economic growth. The study conducted in Colombia shows that combining academic and company learning spaces helps close gaps and promote economic growth by developing intellectual capital, emphasizing decision-making, interorganizational coordination, and knowledge sharing. Quispe Mamani et al. show that education is crucial for financial inclusion in Peru. Financial inclusion is understood as access to quality financial services, essential for leveraging global opportunities for households and companies. The last paper in this section by Broecher and Painter underlines the potential for creating self-determined, healthy, and sustainable forms of working, learning, and living through a transformative community project initiated in Eastern Germany. The investigated initiative integrates components such as self-directed education and unconditional basic income, aiming to foster active civil society and improve conditions for children and young people, with potential for widespread application in rural and urban areas.

## Theme III: empowering communities through entrepreneurship and stakeholder engagement

The final section focuses on two case studies of actions toward supporting specific communities. Naveen et al. concentrated their studies on empowering tribal women through entrepreneurship. The authors argue that particular programs can enhance their economic and social viability. The study suggests increasing government and organizational initiatives to improve women's education and financial capacity to start new enterprises, thereby boosting their decision-making power within their families. The team of Setiawan et al. provides another example based on the scaling up of social entrepreneurship. The authors claim that stakeholder engagement is crucial in Indonesia's state-driven programs to reduce poverty and combine business and social dimensions to improve economic welfare. The research shows that involving state and non-state actors enhances innovation, employment opportunities, and access to capital, ultimately leading to sustainable development and poverty alleviation.

## Conclusion

The results in the Research Topic of articles enable the identification of five directions for further research, namely: (1) the effects of the lack of decent jobs and weak social insurance schemes (see Chen and Carré, 2020); (2) recent technological changes in the world of work related to automation and robotics (see Vos et al., 2023); (3) new forms and models of employment and education responding to megatrends including population aging, climate change, and dissemination of artificial intelligence (see Postepska, 2022); (4) transparent, flexible, and predictable legal employment frameworks development (see Gyulavári and Menegatti, 2022); and (5) development-oriented policies and employment services supporting decent job creation and entrepreneurship (see Shabbir, 2023).
